# Navigating a Delicate Balance: Antibiotic Allergy in the Setting of Renal Infection and Diabetic Ketoacidosis

**DOI:** 10.7759/cureus.106802

**Published:** 2026-04-10

**Authors:** Aun A Bangash, Muhammad A Bangash, Ruben M Meza

**Affiliations:** 1 Internal Medicine, The University of Texas Rio Grande Valley School of Medicine, Edinburg, USA; 2 Internal Medicine, The University of Texas Health Science Center at San Antonio, San Antonio, USA; 3 Internal Medicine, The University of Texas Rio Grande Valley School of Medicine, Valley Baptist Medical Center, Harlingen, USA

**Keywords:** beta-lactam hypersensitivity, computed tomography, euglycemic diabetic ketoacidosis, levofloxacin, lobar nephronia, pyelonephritis, type 1 diabetes melitus

## Abstract

Acute pyelonephritis is a bacterial infection of the renal parenchyma that requires prompt recognition and antibiotic therapy to prevent complications such as sepsis and chronic renal inflammation. We present a case of a 26-year-old woman with poorly controlled type 1 diabetes mellitus and a documented beta-lactam allergy who presented with atypical symptoms, including flank pain without urinary complaints. Laboratory evaluation demonstrated leukocytosis with a negative urinalysis. CT of the abdomen revealed left lobar nephronia with early contralateral involvement of the right kidney. Given her allergy history, she was treated with levofloxacin. During her hospital stay, she developed nausea, vomiting, and metabolic abnormalities consistent with euglycemic diabetic ketoacidosis (eDKA), necessitating intensive care management with dextrose-containing fluids, insulin, and electrolyte correction. The patient improved with appropriate medical management and was discharged in stable condition. This report underscores the importance of individualized antibiotic selection in patients with documented drug allergies, the role of imaging in atypical clinical presentations, and the need for sustained observation for metabolic complications like eDKA in patients with poorly controlled diabetes mellitus and infection.

## Introduction

Acute pyelonephritis is a bacterial infection of the renal parenchyma that is most commonly caused by Escherichia coli. Typically, the presentation of the disease includes systemic symptoms such as fever, chills, hypotension, and tachycardia, alongside genitourinary symptoms such as urinary frequency, dysuria, and flank pain. Prompt administration of antibiotic therapy is essential to minimize complications, such as sepsis, renal abscess, and chronic renal scarring and parenchymal damage [1]. In certain cases, the disease may present with greater complexity, including focal bacterial nephritis (lobar nephronia), a more localized and severe form of infection that may mimic a renal abscess, and subsequent metabolic derangements, including euglycemic diabetic ketoacidosis (eDKA), which can occur without significant hyperglycemia and may delay diagnosis.

Empiric treatment includes third-generation cephalosporins such as ceftriaxone due to their broad coverage against Gram-positive and Gram-negative organisms [1,2]. However, a special situation should be considered in patients with beta-lactam allergy, which is often overreported and does not universally preclude cephalosporin use due to the relatively low rate of true cross-reactivity [3]. Therefore, alternative regimens must be considered that are equally effective and have an acceptable hemodynamic profile in patients with β-lactam allergy. One such alternative is levofloxacin, which is commonly used because it has excellent oral bioavailability, good renal parenchymal penetration, and activity against both Gram-positive and Gram-negative organisms, including uropathogens such as Escherichia, Klebsiella, and Proteus [2]. However, rising antimicrobial resistance and patient-specific risk factors must be considered when selecting appropriate antibiotic therapy, even when choosing alternative agents.

## Case presentation

**Figure 1:**
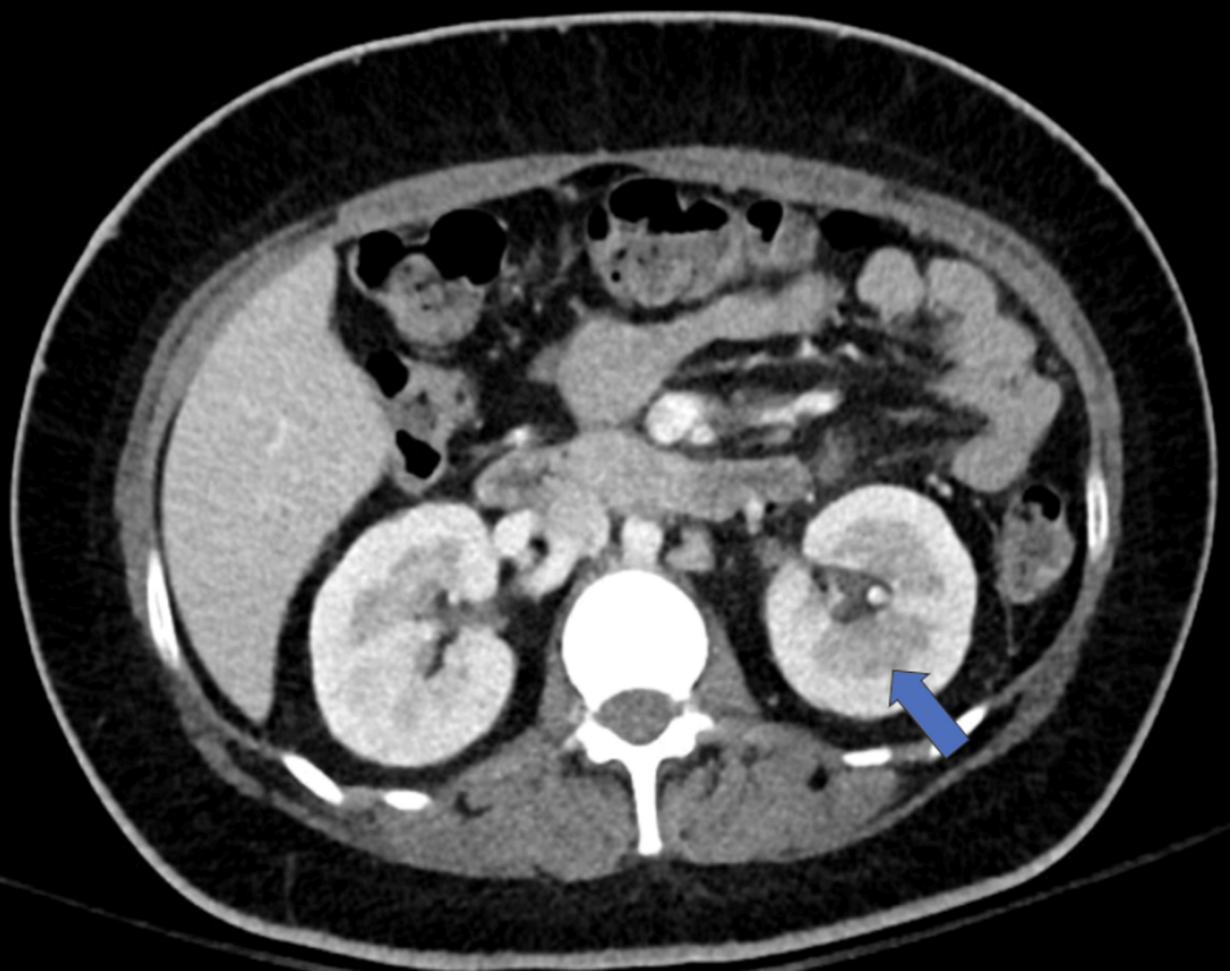
Contrast-enhanced CT of the abdomen Contrast-enhanced CT of the abdomen demonstrating a focal hypodense region within the left renal parenchyma (blue arrow), consistent with lobar nephronia (acute focal bacterial nephritis), and measuring approximately 3.6 cm. There is no rim enhancement or fluid collection, arguing against a renal abscess. Additional subtle inflammatory changes are noted in the contralateral kidney, suggestive of early right-sided pyelonephritis CT: computed tomography

A 26-year-old woman with poorly controlled type 1 diabetes mellitus (HbA1c of 11.9%) and a recent diagnosis of pyelonephritis three weeks prior presented to the emergency department with progressively worsening right flank pain and generalized body aches similar to her previous episode three weeks ago. She denied urinary symptoms such as dysuria, urgency, or frequency. On examination, she was febrile with a maximum temperature of 38.5 °C, tachycardic (heart rate of 116 bpm), and hypertensive (154/89 mmHg). Physical examination revealed bilateral costovertebral angle (CVA) tenderness. Urinalysis was negative for leukocyte esterase and nitrites, while a CBC showed marked leukocytosis with a left shift, suggesting an atypical clinical presentation for an underlying upper urinary tract infection. A CT scan of the abdomen and pelvis with contrast (Figure 1) demonstrated a 3.6 cm lobar nephronia, also known as acute focal bacterial nephritis, of the left kidney and early right-sided pyelonephritis.

The patient's past medical history was significant for angioedema and urticaria following the administration of ceftriaxone during her prior episode of pyelonephritis three weeks ago. Given her recent documented allergy and concern for possible recurrent allergic reactions, levofloxacin, a fluoroquinolone antibiotic, was administered orally once daily at a therapeutic dose of 750 mg. During her hospital stay, the patient began to experience episodes of nausea followed by vomiting and reported diffuse abdominal pain. Due to her clinical presentation and underlying history of poorly controlled type 1 diabetes mellitus, a potential episode of diabetic ketoacidosis was suspected. Repeat laboratory evaluation (Table 1) revealed hyponatremia, hypochloremia, low bicarbonate, elevated anion gap, ketonuria, significant glucosuria, and a serum glucose within normal range.

**Table 1:** Laboratory findings at admission and during eDKA episode Laboratory findings demonstrate Leukocytosis consistent with underlying infection and evolving metabolic derangements during the patient's hospital course. The patient developed an anion-gap metabolic acidosis with significant ketonuria and glucosuria in the setting of normal serum glucose, consistent with eDKA complicating acute pyelonephritis eDKA: euglycemic diabetic ketoacidosis

Parameter	At admission	During eDKA episode	Reference range
White blood cell count (×10⁹/L)	13.7	11.3	4.0-11.0
Sodium (mmol/L)	129	132	135-145
Chloride (mmol/L)	96	103	98-106
Bicarbonate (mmol/L)	18	8	22-28
Anion gap	15	21	08-Dec
Serum glucose (mg/dL)	83	96	70-100
Urine ketones (mg/dL)	Negative	>150	Negative
Urine glucose (mg/dL)	Negative	>1000	Negative
Urine protein (mg/dL)	30	Negative	Negative
Urine nitrites	Negative	Negative	Negative
Leukocyte esterase	Negative	Negative	Negative

Considering the anion gap metabolic acidosis with low bicarbonate, elevated anion gap, and marked glucosuria and ketonuria in the setting of normal serum glucose, along with the sudden onset of gastrointestinal symptoms, the patient was diagnosed with acute pyelonephritis complicated by eDKA. Alternative causes of high anion gap metabolic acidosis, including lactic acidosis and starvation ketosis, were considered but deemed less likely given the presence of significant ketonuria and the clinical context of poorly controlled diabetes.

The decision was made to transfer the patient to intensive care for management and further observation. Interventions included insulin infusion, dextrose-containing fluids, and electrolyte repletion therapy while continuing antibiotic therapy. Subsequently, the patient’s clinical status began to improve, which facilitated transfer to the floor and subsequent discharge.

## Discussion

Acute pyelonephritis is a common upper urinary tract infection that necessitates swift recognition and intervention to prevent clinical complications, including renal abscess formation, sepsis, and long-term renal dysfunction and impairment due to chronic or recurrent episodes of infection. Although classic symptoms include dysuria, urinary frequency, and flank pain with CVA tenderness, atypical presentations may occur, especially in high-risk populations such as those with poorly controlled diabetes mellitus. Consequently, the absence of lower urinary tract symptoms, as seen in our patient, may mask an otherwise classic case of pyelonephritis and delay appropriate management [1].

Diagnostic imaging plays a central role in identifying complicated or atypical clinical scenarios. CT is the imaging modality of choice due to its enhanced sensitivity in detecting focal renal lesions and associated complications [4,5]. Lobar nephronia represents a focal but nonliquefactive infection of the renal tissue that exists within the spectrum of acute pyelonephritis and renal abscess [4]. Therefore, early evaluation is essential, as progression to abscess formation may render antibiotic therapy less effective and may ultimately require invasive intervention.

The selection of antimicrobial therapy must account for pathogen susceptibility and patient-specific risk factors such as prior antibiotic use and documented drug allergies. Third-generation cephalosporins such as ceftriaxone are considered first-line agents for treatment of acute pyelonephritis due to their effective renal penetration, pharmacokinetics, and broad antimicrobial coverage [2]. However, in patients with a history of documented allergic reactions to beta-lactam antibiotics, an alternative agent such as levofloxacin, a fluoroquinolone antibiotic, is used because it also provides excellent renal penetration and antimicrobial coverage. However, as broad-spectrum empiric antibiotics are increasingly used to target infections such as pyelonephritis, rising resistance rates among uropathogens are emerging. Therefore, it is crucial to tailor antibiotic therapy based on culture sensitivities [2].

An additional layer of complexity arises in patients with poorly controlled diabetes mellitus. Infection is a well-known precipitating factor for DKA due to increased insulin resistance and counter-regulatory hormone release, both of which promote ketogenesis [6,7]. This can manifest as eDKA, a variant of DKA characterized by anion gap metabolic acidosis in the presence of normal or mildly elevated serum glucose [6]. Symptoms are often nonspecific but include nausea, vomiting, and diffuse abdominal pain, which may overlap with symptoms of pyelonephritis.

Therefore, in this context as well as other applicable scenarios, the patient’s underlying infection, prior documented drug allergy, and metabolic derangements underscore the importance of maintaining a high index of suspicion for clinically significant complications, which necessitate prompt and effective intervention targeting all contributory factors.

## Conclusions

This report underscores the need to tailor antibiotic therapy in the management of acute pyelonephritis, particularly in patients with a history of drug allergies; alternative agents such as levofloxacin were used successfully in this case. It also showcases how infections in patients with poorly controlled diabetes can lead to serious yet unexpected complications, including eDKA. Identifying these overlapping processes early and addressing both the infection and metabolic derangement simultaneously is key to improving outcomes.
